# An optical pickup enzyme-linked immunosorbent assay (ELISA) with a microfluidic disk†

**DOI:** 10.1039/c8ra01149d

**Published:** 2018-04-18

**Authors:** H. Yoshikawa, M. Yoshinaga, E. Tamiya

**Affiliations:** Department of Applied Physics, Osaka University Suita Osaka 565-0871 Japan yosikawa@ap.eng.osaka-u.ac.jp

## Abstract

We evaluated optical pickup ELISA with an original microfluidic disk that contains eight radially arranged channels, which enable semi-automatic sample loading and washing. This disk-shaped chip composed of acrylic plates was fabricated by CO_2_ laser machining and capture antibodies were immobilized in the channels. After the immunoreaction with antigens and enzyme-linked secondary antibodies, an enzyme-catalyzed nanoaggregation of *o*-phenylenediamine was detected by measuring the reflectivity change of a laser beam focused in the channel. The assay of C-reactive protein (CRP) was successfully performed in a short amount of time (approximately 20 min from CRP loading). The limit of detection was determined to be 2 ng mL^−1^, which is more sensitive as compared with conventional ELISA using microplates.

## Abstract 2

Optical pickup ELISA with an original microfluidic disk, which enable semi-automatic sample loading and washing, was developed. The rapid and sensitive assay of C-reactive protein (CRP) was successfully performed.

## Graphical abstract

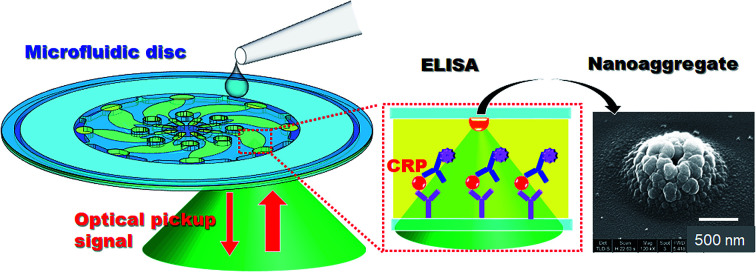

## Introduction

Bioanalyses with microfabricated devices, in which various microstructures for sample delivering, mixing, and signal detecting are integrated, have been actively studied in recent years.^1–4^ Enzyme-linked immunosorbent assay (ELISA) is a powerful and commonly used technique to sensitively detect specific proteins; recently, ELISA with microfabricated chips (microELISA) has attracted much attention. Compared with the conventional ELISA platform using microtiter plates, microELISA is expected to considerably reduce sample and reagent volumes, as well as shorten the reaction (incubation) time for antigen–antibody binding and colorimetric reaction due to high surface-to-volume ratio and limited molecular diffusion in small volume chambers.^5,6^ However, the small reagent volume in a microfabricated device inevitably decreases sensitivity. Therefore, increased immobilization density and control of antibody orientation have been used by some research groups to enhance the efficiency of antigen–antibody interactions (immunoreactions) in small volumes.^7,8^ While these approaches improve assay sensitivity, they are also time-consuming and laborious in most cases. Another approach for signal improvement is the development of sensitive signal detection methods for low sample volumes.

**Fig. 1:**
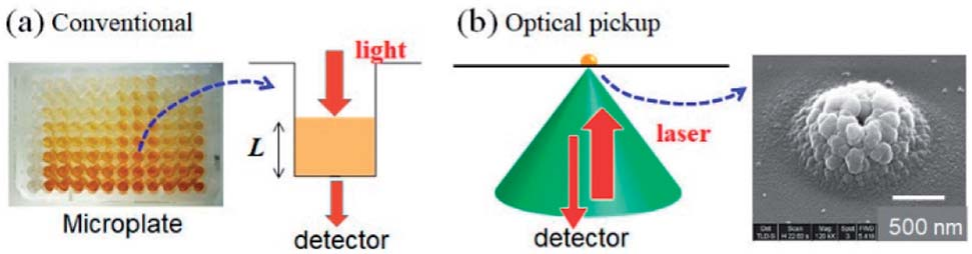
Signal detection schemes of (a) conventional microplate ELISA and (b) optical pickup ELISA.

Let us consider signal detection methods used in conventional ELISA.^9^ Colorimetric assays, including ELISA, are widely utilized in the field of bioanalysis due to their simplicity and high reproducibility. Enzymes such as horseradish peroxidase and alkaline phosphatase, which catalyse the colorimetric change of specific chemical reagents, are usually utilized. Colour changes due to enzymatic reactions are quantitatively evaluated based on optical absorbance measurements. For such colorimetric assays, microtiter plates and plate readers are widely recognized as standard systems, where optical absorbance of the test solution in each well is sequentially and semi-automatically measured. Normally, the degree of colour change (*i.e.* absorbance) of 100–200 μL of the solution is measured to evaluate the concentration of the target protein at the final stage of the ELISA (Fig. 1a). Since absorbance is proportional to the path length of light passing through the solution, reduced solution volume, *i.e.* a decrease in the light path length, results in reduced sensitivity for microELISA. In addition, an error in the solution volume results in error in the result of the measurement.

To overcome this obstacle, several signal detection techniques have been used in previous microELISA studies. Representative methods include detection of luminescence, such as fluorescence and chemiluminescence.^6,10–12^ Luminescence detection is quite sensitive; however, a sensitive photodetector, an excitation light source (for fluorescence), expensive reagents, and a set of optical filters are necessary. Thermal lens detection is also a sensitive method that Kitamori *et al.* applied to their microELISA systems.^13^ However, the thermal lens method requires two laser sources and elaborate optical systems to focus two laser beams at different depths. These detection methods may satisfy the sensitivity requirement, but the detection system used are complicated, bulky, and expensive.

As an innovative way to solve the problems inherent in colorimetric-based ELISAs, we propose the application of optical pickup detection of horseradish peroxidase (HRP), which was recently developed by our group.^14,15^ In principle, this is a method to induce and detect oxidative polymerization (dimerization) of *o*-phenylenediamine (*o*PD) using a single laser beam. *o*PD is a well-known colorimetric reagent used in conventional ELISAs; oxidative polymerization (mainly dimerization) of *o*PD is catalysed by horseradish peroxidase (HRP), and the reaction product (diaminophenazine: DAP) has an intense colour.^9,16^ In conventional colorimetric ELISA, this colour change is evaluated by light absorbance. On the other hand, the optical pickup method detects aggregate deposition due to further oxidative polymerization induced by a focused laser beam (Fig. 1b), which is similar to commercial read-and-wright system of optical discs (CD, DVD, and BD). The detection mechanism was explained in detail in previous reports.^14,15^ The *o*PD reaction occurs with a laser focus of approximately 1 μm in diameter. As a result, the sample volume necessary in this method is on the scale of several nL–pL, which is much smaller as compared with that of conventional ELISA. In addition, signal intensity is not dependent on the optical path length. Furthermore, a low-power visible laser beam and a general photodetector can be used, which allows for simple, compact, and low-cost optical setup. Therefore, optical pickup detection is expected be highly compatible with microELISA applications.

Assessments using commercial pickup devices for CD/DVD/BD suggested that our optical pickup detection has good compatibility with disk-shaped biosensors, which applies fluidic controlled-pumpless microfluidics based on the centrifugal force.^6,17,18^ Although sophisticated disk-shaped chips have been designed and developed to minimize manual labour in ELISA, here we propose a disk-shaped chip for optical pickup ELISA with a simple design to semi-automate the washing process. Inadequate or excess washing harms data quality, and individual difference in the washing process results in error. In this paper, we demonstrated for the first time that ELISA with optical pickup detection can be carried out with an original microfluidic disk, which enables semi-automatic sample loading and washing.

## Experimental

### MicroELISA chip fabrication

**Fig. 2:**
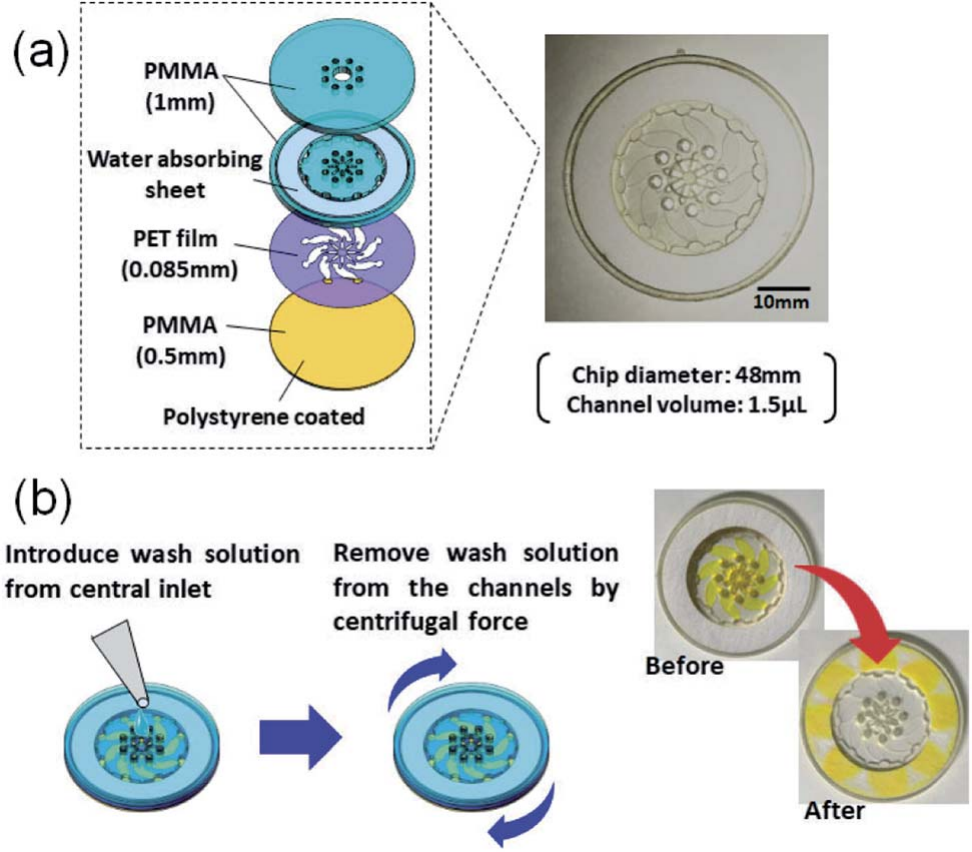
(a) A schematic of microELISA chip (b) washing mechanism by using centrifugal force.

The construct of our microELISA chip is illustrated in Fig. 2. The 48 mm in diameter disk-shaped chip was composed of acrylic (polymethyl methacrylate: PMMA) plates and a polyethylene terephthalate (PET) film. Polystyrene solution (10 wt% in xylene) was spin-coated on one-side (inner side) of the bottom plate to form a hydrophobic surface that immobilizes antibodies. The top plate has a hole in the centre with eight small holes near the centre, which function as solution inlets. A laser beam is focused on the inner surface of the top plate during the optical pickup measurement. The eight corresponding channels were constructed in a PET spacer film (Nitto, No. 5302A) sandwiched by the top and bottom plates. These three plates were fabricated by laser machining (Commax, VD-A3), and were stuck together by a double-sided tape (Nitto, No. 5603); the hole in the centre is connected to each channel. As the chip rotates, a washing buffer solution applied to the hole in the centre flows out through each channel, and is absorbed into a filter paper set at the peripheral of the chip.

## Materials and methods

**Fig. 3:**
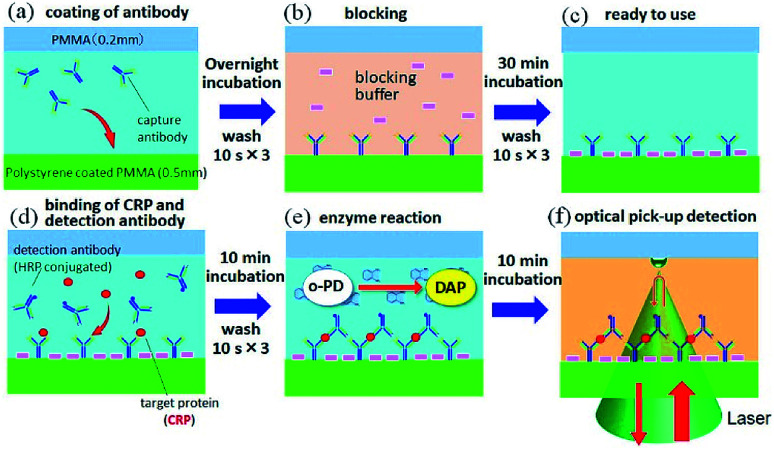
A schematic diagram of assay procedures of the optical pickup microELISA.

Human C-reactive protein (CRP) was chosen as the test target, as it is a representative biomarker protein for cardiovascular disease. There are many previous reports on CRP ELISAs.^19,20^ Anti-human CRP antibodies (abcam, ab8278) were diluted in a coating buffer (abcam, ab210899); this capture antibody solution (10 μg mL^−1^) was dropped into each inlet hole of the chip, which was then naturally introduced into the channels *via* capillary force (Fig. 3a). Following an overnight incubation at 4 °C, capture antibodies were immobilized on the polystyrene coated plate. In this study, physical adsorption of capture antibodies, which is generally used in conventional ELISA with a microtiter plate, was used to demonstrate easy operation and handling of this method. Various immobilization techniques, which have been developed to improve stability, density, and orientation of antibodies, could be useful to reduce the hydraulic effect, as discussed below, and increase the sensitivity.^21^ Residual capture antibodies not immobilized onto the chip surface were rinsed by flowing washing buffer (BD biosciences, 51-9003739) through the channels. This washing process is schematically explained in Fig. 2b. A wash solution was dropped into the hole in the centre of the chip, and the chip was rotated three times at 3000 rpm for 10 s with the spin-coating machine (Oshigane, SC-150) to flow the solution into all eight channels. The same process was also conducted to wash out the blocking buffer (abcam, ab210904) and detection antibodies. Following immobilization of capture antibodies, blocking buffer was introduced into the channels to prevent non-specific binding of target proteins. Capture antibody immobilization was completed by washing out the blocking buffer (Fig. 3b and c).

*o*-Phenylenediamine (*o*PD: Sigma) was dissolved in citrate buffer (pH 4.5) to prepare a 4 mM *o*PD stock solution. This stock solution was mixed with H_2_O_2_ aqueous solution (BioCheck, MB-10024; 0.015% (v/v) H_2_O_2_ in 0.1 M citrate buffer, pH = 5.0) at 1 : 1 ratio just before its use for the HRP specific reaction. Human CRP (BioCheck, BC-1119) was diluted in PBS buffer, and five test solutions (50, 10, 2, 0.4, and 0.08 ng mL^−1^) were prepared. Each solution was mixed with HRP (horseradish peroxidase)-modified anti-CRP (BioCheck, BC-1119) at 1 : 5 just before being introduced into each channel of the chip (Fig. 3d). After a 10 min incubation period, the solution was rinsed with washing buffer. Finally, the *o*PD/H_2_O_2_ solution was poured into each channel (Fig. 3e), and optical pickup detection was performed after 10 min (Fig. 3f).

Optical setup was performed as previously described.^14^ Briefly, the 532 nm line of a DPSS green laser (PHOTOP, DPGL-2100F) was focused onto the interface between the top plate and the *o*PD/H_2_O_2_ solution in a chip channel *via* a 60× objective lens (Olympus, UPlanFLN), as shown in Fig. 3e. The laser power was approximately 2 mW at the focus. A part of the focused laser beam was reflected at the interface, and the intensity of the reflected beam was measured by a photomultiplier (Hamamatsu Photonics, R1166) at a sampling rate of 50 Hz. Temporal change of the reflected laser intensity was recorded in a PC *via* a data acquisition board (National Instruments, USB-6341).

## Results and discussion

In our previous study of glucose detection, a laser beam was focused onto a glass substrate to measure the reflection intensity.^14,15^ To construct microELISA chips using PMMA, the optical pickup signal obtained with a PMMA substrate was evaluated by experiments and computational simulation (see Fig. S1 in ESI†). We found that the PMMA substrate is more suitable for optical pickup detection as compared with glass plates.

**Fig. 4:**
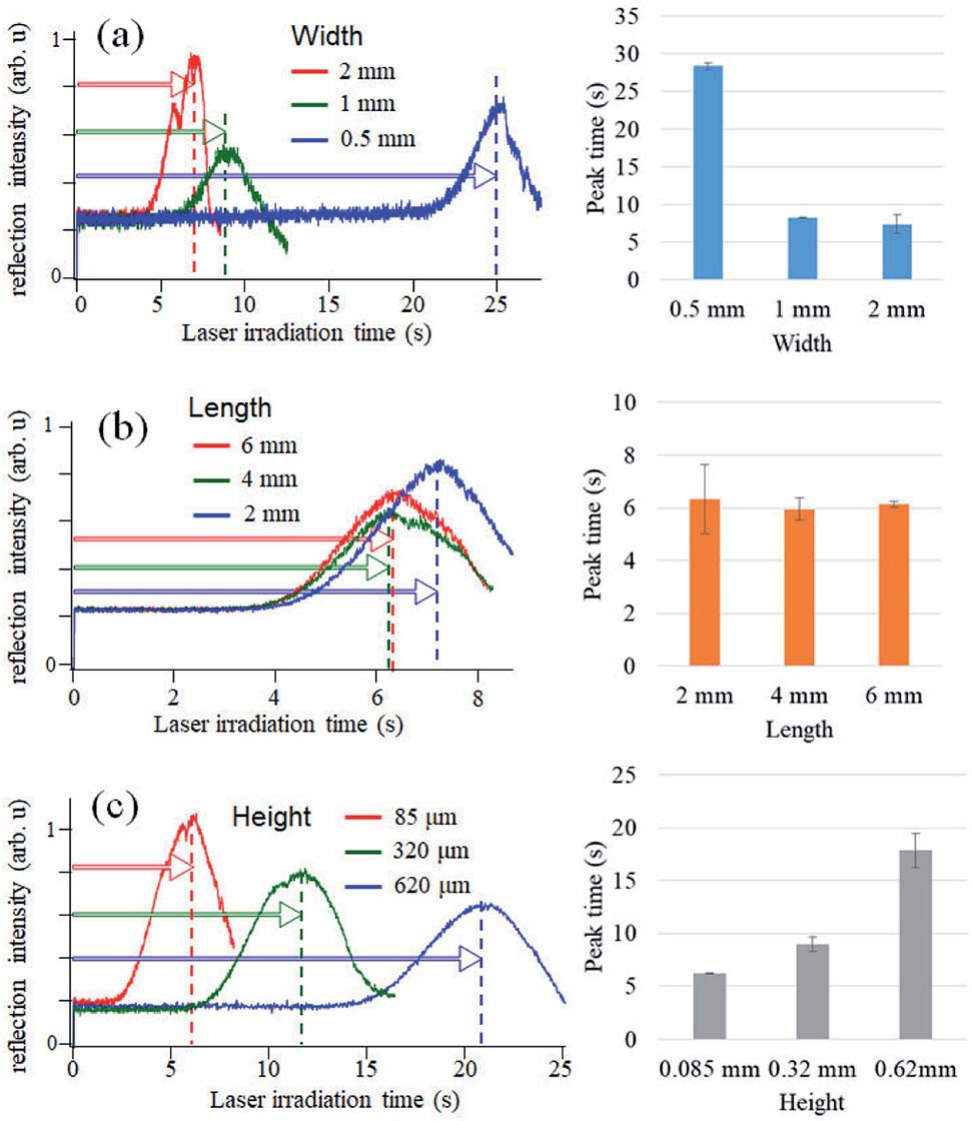
Comparison of optical pickup signal among different channel structures of microELISA chips.

As shown in Fig. 2, our microELISA chip has eight channels to load sample solutions into, and the laser beam is focused for pickup detection. In addition, wash solutions flow through the channels by centrifugal force to rinse out excess proteins. Therefore, channel structure is important for sensitivity and data quality. We evaluated optical pickup signals using test microplates, which has channels with different width, length, and height. Fig. 4 shows the temporal change in the reflection intensity of the focused laser beam, *i.e.* optical pickup signal. The CRP concentration was 100 ng mL^−1^. In all cases, the intensity of the reflected laser beam was first increased, and was then decreased to form a peak in the intensity variation curve. This intensity change is attributed to the formation of a submicron sized aggregate due to the photo-oxidative polymerization of *o*PD induced by the laser absorption in the colored solution, as shown in Fig. 1b. The laser irradiation time at the peak (peak time: *T*_p_) represents the reaction time necessary to generate an aggregate with a certain size, and is dependent on the coloring degree of the solution, that is, the concentration of DAP included in the solution prior to irradiation. In the case of optical pickup ELISA, the degree of the enzyme reaction, *i.e.* the concentration of target biomarkers, is determined from this *T*_p_. Among the three channel widths (Fig. 4a), the 0.5 mm channel yielded the longest detection time (*T*_p_ of approximately 25 s) for the optical pickup signal, whereas the difference between the 1 and 2 mm channels is small. The peak times of the 1 and 2 mm width chip were close to each other as compared with that of the 0.5 mm width chip. Channel width wider than 1 mm would also be appropriate; however, increase in channel width requires greater sample volume. Fig. 4b indicated that channel length has minimal effect on detection result in the length scale of 2–6 mm. However, channel height demonstrated a clear relationship with detection time, as shown in Fig. 4c; an earlier peak time onset was associated with reduced channel height. This indicated that the reaction was enhanced by increased surface-to-volume ratio of the solution-filled channel, since antigen–antibody binding and HRP enzyme reaction occurs at the channel surface of the bottom plate to which antibodies are bound.^22,23^ On the other hand, the surface to volume ratio does not change with the reduction of the channel width. The cause of the width dependence of the sensitivity is under investigation. Hydraulic pressure and/or shear stress on the bottom surface, which depend on the channel shape, may sweep a part of binding antigens/antibodies. Insufficient flow may cause an excess residual of the blocking solution. Thus we believe that the optimal flow rate and the channel shape to maximize the sensitivity exist. Theoretical and simulation analyses are also necessary to discuss deeply such microfluidic properties, but Fig. 4 indicates a channel fabrication guideline for not reducing the sensitivity. Although a channel height shorter than 85 μm is expected to produce faster reactions, the height is determined by the thickness of the commercial PET film, which is used as a spacer to make the channel height. Therefore, we fabricated microELISA chips with a channel width of approximately 3.5 mm and a channel height of 85 μm for the following CRP detections.

**Fig. 5:**
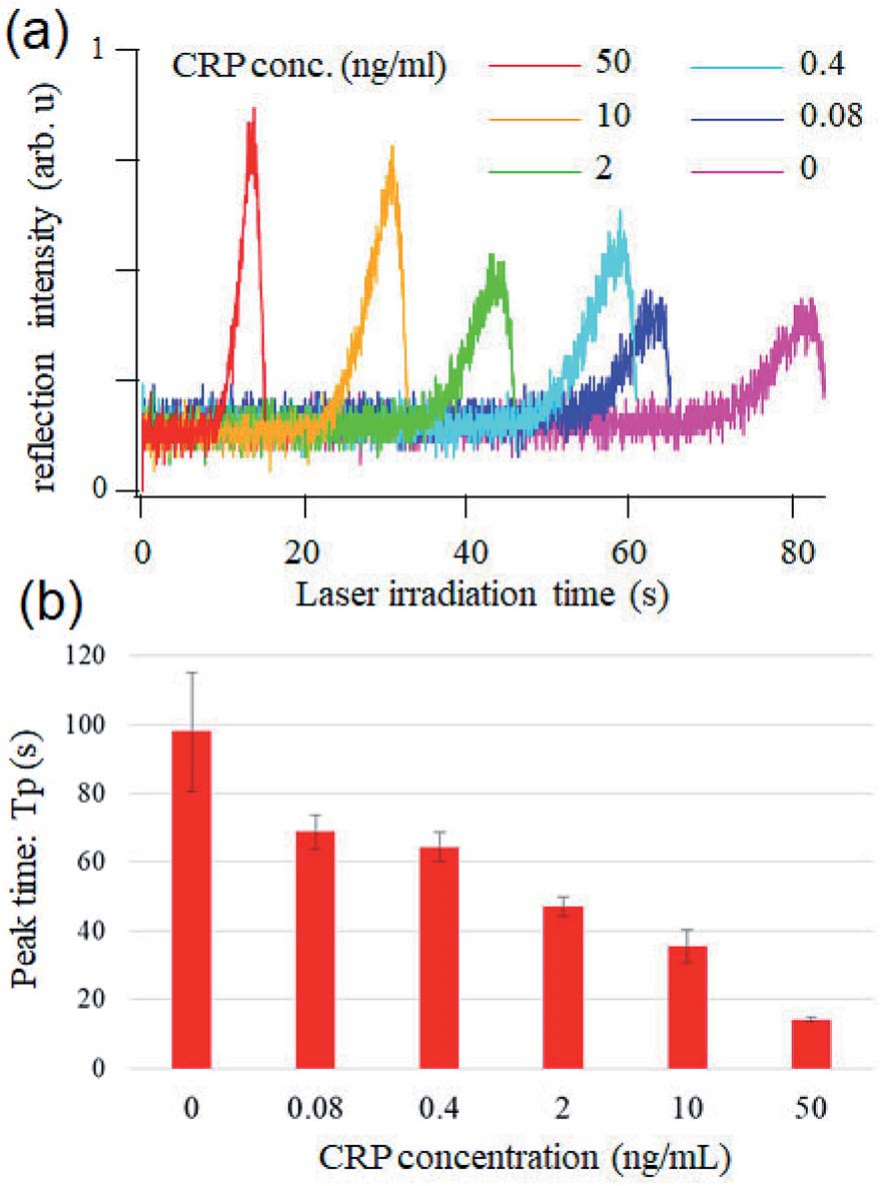
(a) CRP concentration dependence of the optical pickup signal. (b) Peak time *vs.* CRP concentration.

Finally, we performed optical pickup microELISA using the optimally designed chip. Fig. 5a shows the temporal change of the reflected laser intensity measured for each CRP concentration. The intensity of the reflected laser beam gradually increased, and reached its maximum at *T*_p_, which is dependent on CRP concentration. Results indicated that higher CRP concentration is associated with shorter *T*_p_. *T*_p_ was plotted as a function of CRP concentration with a standard deviation of measurements carried out thrice (Fig. 5b). Fig. 5 indicates that pickup signals at 0.4, 2, 10, and 50 ng mL^−1^ are distinguishable and the logarithm of the concentration is roughly proportional to the peak time in this range. The limit of detection (*L*_d_) was estimated to be ∼2 ng mL^−1^ based on the commonly used simplistic way, where *L*_d_ was calculated as a standard deviation of the blank multiplied by 3.^24^ It should be noted that the assay, including the incubation and semi-automatic washing processes, was successfully performed in a short amount of time (approximately 20 min from CRP loading). For comparison, a conventional ELISA of CRP was performed with a microtiter plate (see ESI†). Fig. S2† shows that absorbance at 2 ng mL^−1^ CRP couldn't be distinguished from that of the blank sample and 10–100 ng mL^−1^ CRP was quantitatively detected with a microplate based ELISA using the same reagents and incubation times. This demonstrated that optical pickup ELISA is more sensitive as compared with conventional ELISA. This could be attributed to the high surface-to-volume ratio of the microELISA chamber, which enhances chemical reactions, leading to short antigen–antibody incubation (10 min) and *o*PD reaction (10 min) times.

## Conclusions

Our study showed that the optical pickup method was able to successfully catch ELISA signals from a small volume of sample in a microfluidic chip. Since the volume of the solution used in this study is still much larger as compared with the laser spot size used for optical pickup detection (approximately 1 μm in diameter and 2 μm in depth), sample volumes can be reduced to the sub nL level in principle. In this study, we showcased optical pickup microELISA with a simple disk-shaped chip, and concluded that optical pickup ELISA allows for ultra-small sample volume, as well as rapid and sensitive analyses. Clinical sample testing will be necessary in the next step towards practical applications. The versatility and flexibility exhibited by the optical pickup method should also be noted. Requirement for this method is only a small flat substrate area to focus the laser beam onto. This property extends degrees of freedom in terms of chip design and fabrication. Advanced and sophisticated disk-shaped chips, which have recently been developed,^6,18^ could have good compatibility with the optical pickup method.

## Conflicts of interest

The authors declare no conflicts of interest.

## Supplementary Material

RA-008-C8RA01149D-s001
