# Supplementary material for: An optical pickup enzyme-linked immunosorbent assay (ELISA) with a microfluidic disk
Source: RSC Adv. 2018 Apr 18;8(26):14510–4. doi: 10.1039/c8ra01149d (PMC9082109; doi:10.1039/c8ra01149d)
Supplement: RA-008-C8RA01149D-s001 [file RA-008-C8RA01149D-s001.pdf]

Electronic Supplementary Material (ESI) for RSC Advances.
This journal is © The Royal Society of Chemistry 2018

Supplementary information

An optical pickup enzyme-linked immunosorbent assay (ELISA) with a microfluidic disk

H. Yoshikawa,* M. Yoshinaga and E. Tamiya
Department of Applied Physics, Osaka University, Suita, Osaka 565-0871, Japan
E-mail: yosikawa@ap.eng.osaka-u.ac.jp

Evaluation of glass and PMMA substrates for optical pickup detection

In general, glass is ideal for optical measurements, as it is highly transparent, amorphous, and isotropic as compared with plastic materials. However, flexibility of processing, ease of handling, and robust properties are important for fabrication of biosensor chips. Therefore, we chose PMMA (acryl) as a potential material for manufacturing of microELISA chips; the optical pick-up signal obtained with a PMMA substrate was evaluated by experiments and computational simulation.

The reactive oPD solution (2 mM oPD solution including 10 μM DAP) was dropped onto the glass or the PMMA substrate, and the optical pickup signal was measured under the same condition (Fig. S1a). Although peak times were almost identical between the glass and the PMMA substrate, signal contrast (difference between minimum and maximum intensities) of the PMMA substrate was higher as compared with that of glass. Our result demonstrated that the PMMA substrate is suitable for optical pick-up detection.

The difference in optical pick-up signals between the glass and the PMMA substrate was simulated by the finite-difference time-domain (FDTD) method. The simulation was performed using the Rsoft Fullwave software. The 2-dimensional calculation model, in which an aggregate on glass or PMMA substrate was illuminated by a Gaussian laser beam (wavelength 532 nm), was designed as shown in Fig. S1b. By changing the size of the aggregate, different models were produced; simulation was performed for each model. As shown in Fig. 1b in the main text, the aggregate structure is in the shape of an inverted bowl. In the simulation, we assumed that the shape of the aggregate is part of a 1 μm sphere, and that growth of the aggregate was represented by changing the degree of exposure of the sphere on the glass surface (Fig. S1b). The simulation result (Fig. 1b) showed that reflection intensity of the PMMA plate was lower as compared with that of glass at $t = 0$; this relative position was changed at peak time. This suggested that the signal contrast in the optical pickup of PMMA is higher as compared with that of the glass plate. This was consistent with experimental results. Refractive index of the oPD aggregate was fixed (n-ik: $n = 1.8$, $k = 0.3$), while the size (diameter and height) varied based on calculations. However, in actual

experimental conditions, refractive index may change due to growth of the aggregate, i.e. increased degree of polymerization. In addition, the reflection (back scattering) intensity is dependent on the shape of the aggregates. Those dynamic changes in size, shape, and refractive index could influence the curve shape in the actual experiment.

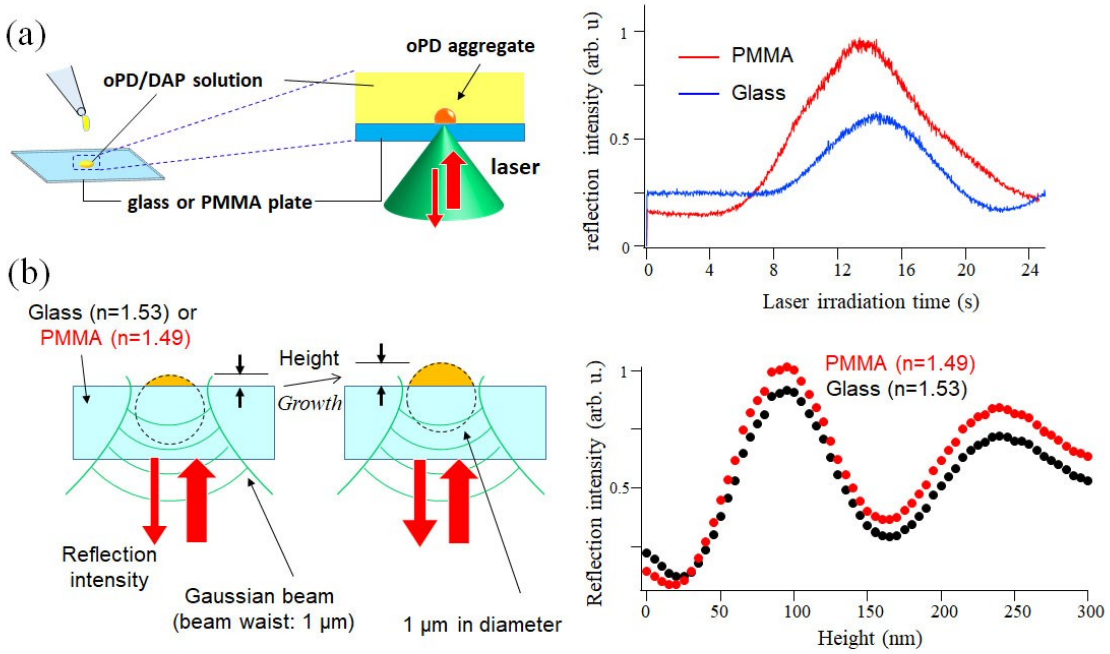

Fig. S1 (a) Temporal changes of reflection intensities of the focused laser beam (optical pickup signal) using glass and PMMA substrates. (b) FDTD simulation results and models. The reflection intensity of a Gaussian beam is plotted as a function of aggregate height. The simulation was performed for glass (n = 1.53, black marker) and PMMA (n = 1.49, red marker). The aggregate shape is modeled as a part of a sphere whose diameter is 1 μm.

Conventional microplate ELISA of CRP

In conventional ELISA, capture antibodies are immobilized onto the surface of each well in a microplate (microtiter plate), and colorimetric reactions are evaluated by the absorbance of the solution in the well. To compare with experimental results of optical pickup microELISA, a conventional ELISA of CRP was performed with a commercial ELISA kit (BioCheck, BC-1119). Same chemical and biological reagents were used for both ELISAs. Incubation times of the antigen-antibody interaction (10 min) and the oPD reaction (10 min) were also the same between the two methods. The washing processes for optical pickup microELISA followed the conventional methods

used for the microplate. The assay result (Fig. S2) indicated that sensitivity of conventional microplate ELISA was between 10 - 100 ng/mL CRP, which was lower than that of optical pickup ELISA. It should be noted that incubation times of the antigen-antibody interaction and the oPD reaction were much shorter as compared with those described in the standard protocol of the ELISA kit (antibody interaction: 45 min, oPD reaction: 30 min). Our study indicated that optical pickup microELISA enables rapid microanalysis using minimal amount of conventional reagents.

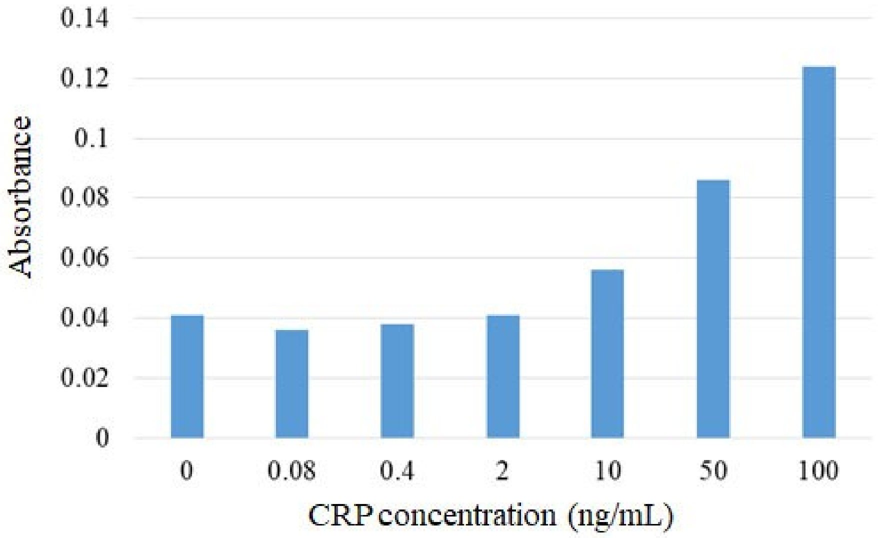

Fig. S2 ELISA results performed using a conventional microplate.
